# Hematopoietic Stem Cell Transcription Factors in Cardiovascular Pathology

**DOI:** 10.3389/fgene.2020.588602

**Published:** 2020-10-16

**Authors:** Sushmitha Duddu, Rituparna Chakrabarti, Anuran Ghosh, Praphulla Chandra Shukla

**Affiliations:** School of Medical Science and Technology, Indian Institute of Technology Kharagpur, Kharagpur, India

**Keywords:** hematopoeitic stem cell, transcription factors, epigenetics (DNA methylation), histone modifications, cardiovascular diseases

## Abstract

Transcription factors as multifaceted modulators of gene expression that play a central role in cell proliferation, differentiation, lineage commitment, and disease progression. They interact among themselves and create complex spatiotemporal gene regulatory networks that modulate hematopoiesis, cardiogenesis, and conditional differentiation of hematopoietic stem cells into cells of cardiovascular lineage. Additionally, bone marrow-derived stem cells potentially contribute to the cardiovascular cell population and have shown potential as a therapeutic approach to treat cardiovascular diseases. However, the underlying regulatory mechanisms are currently debatable. This review focuses on some key transcription factors and associated epigenetic modifications that modulate the maintenance and differentiation of hematopoietic stem cells and cardiac progenitor cells. In addition to this, we aim to summarize different potential clinical therapeutic approaches in cardiac regeneration therapy and recent discoveries in stem cell-based transplantation.

## Introduction

**FIGURE 1:**
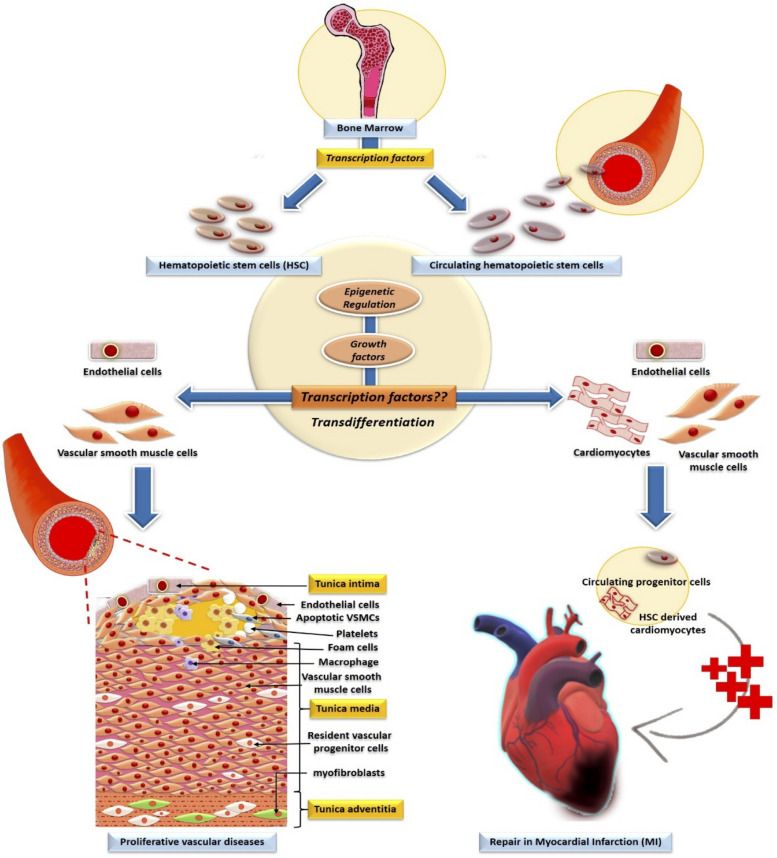
Involvement of transcription factors and their epigenetic modification in the development of HSC. Bone marrow-derived-HSCs and circulating HSCs transdifferentiate to non-blood lineage cells in vascular pathology and repair of the cardiac tissue. Whereas the role of transcription factors in the trans-differentiation of HSC to cardiac and vascular cells are undefined.

Vast gene networks modulating normal physiological and developmental processes as well as disease progression in humans is controlled by more than 1600 molecular mediator proteins known as transcription factors. They play the role of key regulators of the gene transcription processes by specifically binding with enhancer/promoter/silencer regions of the chromatin via their DNA-binding domains to modulate their target gene/s. These factors can act individually as well as in a combinatorial manner to influence the expression of single or multiple target genes in cells to create a colossal spatiotemporal gene regulatory network. The quest for these factors led to numerous studies in which their role has been extensively investigated in all the cell types which were involved in developmental pattern formation, immune response modulation, and inflammatory diseases (Latchman, 1993; Rahman and MacNee, 1998; Campbell et al., 2000; Liang et al., 2001; Coffer and Burgering, 2004; Mudter et al., 2005; Rutenberg et al., 2006; Vaquerizas et al., 2009; Dejean et al., 2010; Wilson et al., 2011; Singh et al., 2014; Lambert et al., 2018, Lammers et al., 2020). These studies have revealed several transcription factors that are crucial in synchronizing the earliest development of the embryo by balancing the differentiation of the stem cells present in the 3 germ layers. With the advancement of developmental as well as stem cell biology the phenomenon of hematopoiesis started to make a significant mark in understanding the critical role of transcription factors in these crucial processes. Hematopoietic stem cells (HSCs) are vital progenitors of the hematopoiesis, which gives rise to both lymphoid and myeloid cell lineages from the primitive mesodermal layer in the early embryonic stages of development (Kehrl, 1995; Ross et al., 2000; Takakura et al., 2000; Zhu and Emerson, 2002; Qian et al., 2007; Miranda-Saavedra and Göttgens, 2008; Fong and Tapscott, 2013; Takahashi and Yamanaka, 2016; Ng and Alexander, 2017). These crucial HSCs are a small subpopulation of bone marrow cells, that bear the hallmark of pluripotency and self-renewal capacity (Schofield, 1978; Miranda-Saavedra and Göttgens, 2008; Ng and Alexander, 2017). Apart from being the master regulators of the HSCs, these transcription factors are also involved in the development of other organs, such as heart and vascular systems as well as in diseases. Consequently, any mutation/dysfunction of hematopoietic transcription factors in the lymphoid cells has been shown to cause diseases like leukemia, failed embryonic, and immune system development (Deguchi and Gilliland, 2002; Yang and Karsenty, 2002; Koschmieder et al., 2005; Rosenbauer and Tenen, 2007; Mullighan et al., 2009). Furthermore, mutations in these transcription factors resulted in cardiovascular diseases (CVDs) like coronary artery disease, cardiac hypertrophy, and congenital heart diseases involving conduction abnormalities and cardiac malformations (Schott et al., 1998; Ikeda et al., 2002; Bhagavatula et al., 2004; Akazawa and Komuro, 2005; Kohli et al., 2011; Ang et al., 2016). In response to the cytokines and growth factors released in several CVDs, HSCs and circulating HSCs have the potential to conditionally differentiate into cardiomyocytes and vascular cells (Sata et al., 2002) (Figure 1). Another characteristic of the transcription factors is that these master regulators are either epigenetically modified or control the target chromatin regulator epigenetically in both hematopoietic developments as well as in disease progression. This review also focuses on clinical aspects of bone marrow HSCs transplantation in replacing the lost cells due to CVDs. In recent years, HSC transplantation has become one of the most effective therapies to promote cardiac regeneration post-ischemia, a major cause of high mortality rates in CVDs (Mueller et al., 2018). However, the therapeutic potential of stem cell transplantation is still a debated topic due to limitations in the stem cell isolation, *in vivo* propagation, and trans-differentiation, besides social, ethical and other concerns regarding its acceptance and usage (Liao and Tse, 2013). Henceforth, the clinical treatment of CVDs started to undertake newer strategies to overcome the limitations of stem cell transplantation-based therapy, such as extracellular vesicle (EV)–based therapy currently being explored widely which entails a specific transfer of bioactive molecules like RNA, micro-RNA, lipids, and proteins to stimulate cardiac tissue regeneration post CVD induced damage (Amosse et al., 2017).

Understanding the etiology of a disease requires the elucidation of its underlying pathophysiology. Due to the crucial role of transcription factors in directly modulating the gene expression, which in turn contributes to the disease pathophysiology, these factors possess the potential to become promising therapeutic targets in several CVDs. In this review, we specifically focus on transcription factors that are involved in the differentiation and development of both HSCs and cardiovascular cells, with an emphasis on their role in various CVDs. Additionally, we have also tried to shed light on the current advances in stem cell-based therapies for CVDs.

## Common Transcription Factors in HSC Development and Cardiovascular Pathology

Transcription factors play an important role in the development and proliferation of progenitor cells, and their differentiation into specific lineages. We have selectively focused on the transcription factors which are equally pivotal for both the maintenance and differentiation of HSCs, as well as involved in embryonic heart development and diseases (Table 1). It becomes more pertinent because, in most of the diseases including CVDs, there always is some degree of fetal gene reactivation that drives remodeling and future phenotypic modulation. This fetal gene program closely resembles the active genes of stem cells and stem cell-like resident cells. Hence, a comprehensive understanding of these transcription factors may help us in shedding light upon the potential target genes involved in the modulation of the ‘cardioprotective’ effect in patients of CVDs.

**TABLE 1:** Transcription factors and their roles in HSC and cardiovascular development.

Transcription factors	Hematopoietic stem cell development	HSC lineage development and malfunction	Cardiovascular development	Cardiovascular Pathology	Heart regeneration	References
GATA family	Proliferation and differentiation of HSC	+++	Embryonic heart development	Pro-hypertrophic factor	+++	Hautala et al., 2001; Ling et al., 2004; Pikkarainen et al., 2004; Rysä et al., 2010; Singh et al., 2010; Fukata et al., 2013; Al-Maqtari et al., 2017; Yoon et al., 2018; Zhuang et al., 2019
SCL	Development and differentiation of HSC to erythroid and megakaryocyte lineage	++	Vascular system development	Pro-angiogenesis factor	+	Visvader et al., 1998; Hall et al., 2003; Lazrak et al., 2004; Bussmann et al., 2007; Porcher et al., 2017; Tobin et al., 2017
Mef2	Maintain regulatory mechanism of megakaryocyte	+	Embryonic heart development	Pro-hypertrophic factor	+++	Hall et al., 2003; Bussmann et al., 2007; Wilker et al., 2008; Gekas et al., 2009; Pon and Marra, 2016; Al-Maqtari et al., 2017; Tobin et al., 2017; Wei et al., 2017
NFAT	Lymphoid cell development	+	Cardiac muscle and vascular wall development	Atherosclerotic plaque development	++	Graef et al., 2001; Bushdid et al., 2003; Hogan et al., 2003; Chang et al., 2004; Liu et al., 2004; Sanna et al., 2005; Müller et al., 2009; Chen et al., 2017
LMO2	Key regulator of hematopoiesis	++	Endothelial differentiation	Crucial for angiogenesis Tumor angiogenesis	+	Neale et al., 1995; Yamada et al., 2000, 2002; Gering et al., 2003; Terano et al., 2005; Meng et al., 2016; Sincennes et al., 2016
Forkhead box family	Critical role for HSC self-renewal	+++	Vascular development	Regulate several cardiac diseases	+	Kume et al., 2001; Seo et al., 2006; Miyamoto et al., 2007; Hou et al., 2015; Xin et al., 2017; Inman et al., 2018; Song et al., 2019; Whitesell et al., 2019; Zhou et al., 2019
Runx family	Maintaining definite hematopoiesis	+++	Heart and vascular development	Cardiac ischemia	+	Wang et al., 1996; Gattenlöhner et al., 2003; Lluri et al., 2015; de Bruijn and Dzierzak, 2017; Mevel et al., 2019; Koth et al., 2020
ETS family	Hematopoietic development Megakaryopoiesis	+++	Embryonic vasculogenesis and angiogenesis	Vascular inflammation and remodeling	++	Hromas et al., 1993; Oettgen, 2006; Lacadie and Zon, 2011; Bosman et al., 2015; Liu et al., 2015; Tejerina et al., 2017

*‘+’ indicates the weight of contribution of transcription factors in HSC lineage development and malfunction as well as in heart regeneration (+++ major, ++ moderate, + low).*

### GATA Family

The GATA transcription factors belong to the family of double Zinc finger domain-containing proteins (CysX2-CysX17-CysX2-Cys) and are characterized by their binding specificity toward the consensus DNA sequence (A/T) GATA(A/G). The amino acid sequence is quite similar to the zinc finger, the C-terminal of the zinc finger is responsible for binding at the DNA-sequence and the N-terminal is required to bind at the friend of GATA (FOG) which maintains the stability and specificity of the DNA binding (Kohli et al., 2011). GATA along with other transcription factors like SCL-1, LMO2, LYL1, and E-proteins forms the transcription complex and regulates the differentiation process of HSCs. Certain post-translational modifications and epigenetic variations further fine-tune the activity of the GATA proteins. The GATA family consists of 6 members. The GATA 1, 2, 3 are mainly involved in the proliferation and differentiation of HSCs and regulate the process of hematopoiesis. The other member’s like GATA 4, 5, 6 are well known for their activities in the embryonic heart development and proliferation of cardiac cells (Pikkarainen et al., 2004). Gata2 is mainly expressed in the HSCs and progenitor cells and regulates the hematopoietic process, and the deletion of Gata2 in mice leads to defects in the hematopoietic process resulting in severe anemia and death. *In vitro* deletion of Gata2 in the embryonic stem cells results in improper response to stem cell factor and augmented apoptosis of the cells. Haploinsufficiency of Gata2 in mice severely affects the number and expansion of HSCs mainly in aorta gonad-mesonephros, the first place where HSCs expand during development (Ling et al., 2004). Gata2 is also well known to maintain the proliferation rate of HSCs in bone marrow (BM). Heterozygous Gata2 null mice show reduced functional HSC numbers and high cellular quiescence and apoptosis (Tipping et al., 2009). Gata3 on the other hand is mainly involved in the differentiation and survival of T helper 2 cells. It is mostly expressed in common lymphoid progenitor cells and differentiates them into T-cells while inhibiting their differentiation into B-cells (Tindemans et al., 2014). Gata1 is essential for the development of megakaryocytes, and deficient megakaryocytes exhibit several abnormal characteristics such as reduced polyploidization, hyperproliferative phenotype, and, reduced expression of megakaryocyte specific genes (Vyas et al., 2000).

Other members of the GATA family, such as GATA4-6 are involved in the development of the mesoderm and endoderm whereas, GATA6 plays a crucial role in mammalian cardiac development. GATA4 and 6 are identical in their primary DNA sequences and share partial sequence motifs. Although most of the functions of these two are redundant during development and in regulating the response to hypertrophic stimuli, individually they are important for the maintenance of cardiac homeostasis and remodeling post-injury (Pikkarainen et al., 2004). Gata4 is reported to be one of the most active Gata-binding factors in the developing heart. Well-characterized functions of Gata4 are its involvement in the differentiation of visceral endoderm and ventral morphogenesis. The deletion of the GATA4 in transgenic mice results in embryonic death, due to improper heart tube formation (Kuo et al., 1997). Studies have reported that any mutation or deletion of the Gata4/5 leads to cardiac disorders including aberrations of the cardiomyocyte proliferation and maturation of the heart chambers (Singh et al., 2010). Gata4 increases the differentiation of embryonic stem cells to beating cardiomyocytes, and inhibiting Gata4 *in vitro* by antisense technology, averts differentiation of the cardiac myocytes, and activates the apoptotic pathway. In contrast to the loss-of-function, *in vivo* up-regulation of Gata6 and concomitant silencing, the function of Gata4 has minimal effects on the differentiation of cardiac myocytes suggesting Gata6 is necessary for maintenance and survival of cardiomyocytes. In addition to the cardiomyocyte-specific functions, a recent study described the role of Gata6 in a cell-specific manner in vascular smooth muscle cells (VSMCs) and endothelial cells (ECs). The study concluded that the deletion of Gata6 in ECs decreased the proliferation of the VSMCs and attenuated the neointima formation after injury, compared to the wild type littermates (Zhuang et al., 2019). The endothelial Gata6/PDGF pathway is responsible for regulating the proliferation rate of VSMCs and also controls the neointima formation. On the contrary, Gata6 deletion in VSMCs changes their phenotype from contractile to proliferative synthetic phase and induces the formation of neointimal hyperplasia post-injury (Zhuang et al., 2019). Interestingly, these studies support the hypothesis of the reversion of adult cardiac/vascular cells to a type of cell that possesses some of the features of primitive stem cells like HSCs.

GATA4 and 6 are also well known to be involved in the hypertrophic phenotype of the heart. Both *in vivo* and *in vitro* studies support the contribution of these transcription factors in regulating the hypertrophic condition (Hautala et al., 2001; Liang et al., 2001; van Berlo et al., 2014). Multiple hypertrophy-promoting genes like α-Myosin Heavy Chain (α-MHC), β-Myosin Heavy Chain (β-MHC), Atrial Natriuretic Factor (ANF), Brain Natriuretic Peptide (BNP), myosin light chain-1/3 (MLC-1/3) are well controlled by GATA4. Overexpression of Gata4 and 6 in the heart is enough to stimulate cardiomyocyte hypertrophy, characterized by an increase in cell surface area and protein accumulation (van Berlo et al., 2014). *In vivo* administration of arginine-vasopressin (AVP) in rats causes a significant 2.2-fold upsurge in Gata4 binding in the ventricles under pressure-overload. Treatment with endothelin receptor blocker, Bosentan inhibits Gata4 binding activity and prevents pressure-overload (Hautala et al., 2001). Gata4 is a known hypertrophy inducing factor, however, few studies have also described its protective role in hypertrophy and post-infarction remodeling. Experimental induction of myocardial infarction by left anterior coronary artery ligation in rats causes an increase in the DNA-binding potential of Gata4 within 2 weeks. Pre-ligation adenoviral transfer of Gata4 causes a significant reduction in the infarct size while increasing the ejection fraction. The cardioprotective role was determined by a decrease in the rate of apoptosis, an increase in the c-kit^+^ stem-like cells, which help in regenerating the functional myocardium, and the number of capillaries was significantly increased in the Gata4 pre-treated hearts compared to the untreated. Microarray analyses have revealed that Gata4 overexpression in the heart in turn increases the expression of CCAAT/enhancer-binding protein (C/EBP) β and C/EBP δ, well known for their involvement in regulating the extracellular matrix genes in cardiomyocytes (Rysä et al., 2010). These results summarize a protective role of Gata4 in myocardial infarction remodeling and also propose Gata4 based gene transfer as a therapeutic approach for dealing with heart failures. Overall, the findings indicate Gata as the primary transcription factor expressed in HSC and its critical involvement in hematopoiesis and megakaryopoiesis. Moreover, Gata has significant regulatory role in developing hearts as well as a pro-hypertrophic factor in adult hearts.

### Myocyte Enhancer Factor 2

Myocyte enhancer factor 2 (Mef2) are a group of proteins that belong to the family of transcription factors named MCM1-agamous-deficient-serum response factor (MADS), consisting of four distinct members Mef2 A, B, C, D. Mef2 has a wide variety of functions in different cells including cardiomyocytes, HSCs, cardiac and skeletal muscles. Mef2c in comparison to the other family members is preliminarily expressed and involved in the mouse embryogenesis, hematopoiesis, and differentiation of cardiomyocytes (Gekas et al., 2009). It is differentially expressed in the progenitor cells and regulates hematopoietic development. Studies have reported that Mef2c is abundantly expressed in the HSCs and common lymphoid progenitor cells (CLPs). Whereas the expression declines when common myeloid progenitor cells (CMPs) differentiate into much committed forms like granulocyte myeloid progenitors and megakaryocyte erythroid progenitors. Several knockout mice models were generated to understand the role of Mef2c in the differentiation of CMPs (Potthoff and Olson, 2007). The systemic deletion of Mef2c leads to embryonic death at E9.5 due to several cardiovascular defects. However, Cre-*loxP* transgenic mice (Mef2c^*f/*–^) when used to determine the role of Mef2c in monocyte differentiation, the selective deletion of this transcription factor reveals no significant effect on the development and maintenance of myeloid progenitors (Stehling-Sun et al., 2009). Interestingly, *in vivo* deletion of Mef2c in bone marrow results in a minor drop in the number of granulocytes compared to the wild type. Overexpression of this factor in immature bone marrow cells leads to a severe decrease in granulocyte numbers (Cante et al., 2013). Mef2c deficient B-cells failed to respond to lipopolysaccharide and had lower immunoglobulin G1 (IgG1) compared to controls. This emphasizes the critical role of Mef2c in B-cell activation upon antigen stimulation (Wilker et al., 2008). Mef2C^*f/f*^ mice cross with Vav-cre^+^(expression of Mef2c is deleted from all the hematopoietic progenitor cells), resulting in a substantial decrease in the peripheral B-cell count without having much effect on the T-cell count. Mef2c^*f/f*^ when crossed with CD19-Cre mice (that specifically knocks out the expression of Mef2c in B-cell), the resulting mouse shows defects in pre-B-cell development and due to defects in the IgM induced activation by cyclin-dependent mechanism (Pon and Marra, 2016). These studies suggest an important role of Mef2c in the B-lymphoid lineage development and survival along with B-cell activation upon antigen stimulation.

Mef2c is well known for its contribution during the embryonic cardiac development. Its deletion causes failure in the right ventricular formation and arrests of cardiac looping during embryogenesis. Mef2c binds to the AT-rich regions of the DNA sequence and activates numerous cardiac genes such as α-MHC, muscle creatine kinase, MLC1/3, cardiac troponin T&C, and several others (Desjardins and Naya, 2016). Mef2c also has a central involvement in cardiac hypertrophy and remodeling after heart failure. The DNA-binding activity of Mef2c increases in the pressure overload-induced cardiac hypertrophy in mice. The hypertrophic signaling pathway which activates Mef2c, principally includes phosphorylation of p38 MAPK, ERK5 also known as MAPK1 and PI3K-AKT pathway. Both *in vivo* and *in vitro* studies show the involvement of all three signaling pathways under hypertrophic-stimulation. Mef2c along with other transcription factors such as Gata, Tinman, and bHLH is vital for the expression of numerous genes required to maintain cardiomyocyte contractility (Potthoff and Olson, 2007; Wei et al., 2017). Calcium signaling plays a critical role in the generation of the hypertrophic phenotype in cardiomyocytes. Mef2c modulates the calcium pathway indirectly via epigenetic modifications. Its activity is tightly regulated by histone deacetylase II (HDAC II), which forms a repressive protein complex around the Mef2c-dependent genes. However, several calcium-controlled protein kinases, like protein kinase D (PKD) and calcium calmodulin-dependent protein kinases (CaMKs) phosphorylate and deactivate the HDAC II; this subsequently results in the activation of all Mef2c-dependent genes responsible for inducing hypertrophy (Passier et al., 2000). A recent study reported that acetylation of Mef2c is required to maintain the pathological phenotype of hypertrophy. *In vivo* administration of 8MI, a synthetic molecule that interferes with the Mef2 co-regulator binding recovers the cardiac hypertrophy in mice subjected to 3 different stress without affecting the phenotype that is observed in cultured cardiac cells (Wei et al., 2017). Mef2c is also known for its involvement in cardiac remodeling. Experimentally induced heart failure in the Mef2 sensor mouse model (MEF2-LacZ transgenic sensor mouse) by transverse aortic constriction (TAC) for 6 weeks and subsequent treatment by beta-blocker for 4 weeks with atenolol lead to the recovery of cardiac function and a decline in the overall activity of Mef2 was observed. Transcriptome analysis from the left ventricular tissue revealed a total of 65 differentially expressed genes under the TAC condition. These 65 genes when mapped in cardiomyocytes showed repressed Mef2 expression. The transcriptome studies discovered Rarres2 as a novel target of Mef2. This study remarks on the role of Mef2 in cardiac remodeling and identifies several genes involved in heart failure, which can be used as markers for disease diagnosis and therapeutics (Tobin et al., 2017). Mef2c plays a critical role in early hematopoietic development and B-cell lineage commitment. In addition, Mef2c is important for embryonic heart development and controls the expression of cardiac-specific genes under remodeling.

### Stem Cell Leukemia Gene/T-Cell Acute Lymphoblastic Leukemia Gene 1 (Scl/Tal1)

The basic helix-loop-helix (bHLH), Scl, or Tal1 is well known for its essential role in embryonic and adult hematopoiesis and is also responsible for embryonic vascular development. First identified as an oncogene, SCL is involved in the chromosome translocation of T-cell acute lymphoblastic leukemia. It forms a complex with LIM domain only protein-2 (LMO2) and maintains the lineage development and diseases. Porcher et al. (2017) reported that Scl knockout murine model reported death on day 9 of the embryonic development due to a lack of primitive erythropoiesis and myelopoiesis. In the adult mouse model, the complete absence of hematopoietic lineage was observed in conditionally deleted Scl. The restriction deletion of Scl in mice by Cre-*loxP* technology shows the involvement of this transcription factor in the regulation of the embryonic hematopoiesis. *In vitro* and *in vivo* deletion of Scl perturbs the formation of progenitor cells in the megakaryopoiesis and erythropoiesis lineage process. The deletion of Scl in BM of transgenic mice resulted in a lymphoid pool consisting of only myeloid cells, whereas the immature progenitors were incapable of producing erythroid and megakaryocyte cells (Hall et al., 2003). All these studies indicate a central role of Scl in the initial differentiation of the progenitor cells and mesodermal cell fate. It has also been reported to play a very significant role in vascular network formation after angioblast. Several animal studies have shown that deletion or any mutation in the Scl expression disrupts the normal development of the vasculature. For instance, the functional loss of Scl in Xenopus leads to the disorganization of major blood vessel formation (Ciau-uitz et al., 2014); whereas in mouse embryos, the loss causes interrupted extraembryonic angiogenic remodeling (Visvader et al., 1998). Overexpression of Scl in the zebrafish embryo causes the mesoderm to differentiate into the blood and endothelial tissues at the expense of the myocardial and somitic tissues. During the early myocardium morphogenesis, the migration of the endocardial precursors to the site of the tube is crucial for the proper formation of the cardiac valve and septa. Scl mutation in zebrafish embryos severely affected the migration of the progenitors, which subsequently lead to the accumulation of the ECs at the ventral pole of the heart (Bussmann et al., 2007). Hypoxia is well known to modulate several transcription factors. A pro-angiogenic stimulus such as hypoxia induces Scl turnover, which is tightly regulated by the phosphorylation of mitogen-activated protein kinase (MAPK) (Tang et al., 2002). *In vitro* studies have shown the involvement of Scl in the formation of new blood vessels, i.e., angiogenesis. ECs originating from micro-vessels express more Scl compared to the BM derived ECs. SCL overexpression in human umbilical vein endothelial cells (HUVECs) results in more capillary tube-like formation compared to the empty vector or mutant dysfunctional SCL. In mice with Matrigel implants containing adenovirus expressing wildtype (WT) Scl enhances the vascularization process in comparison to the mutated Scl (Lazrak et al., 2004). These results indicate the key involvement of Scl in EC motility and in regulating the post-natal angiogenesis. In conclusion, Scl is essential for hematopoiesis, maturation of both megakaryopoiesis and erythropoiesis, as well as contributes in vascular remodeling.

### Nuclear Factor of Activated T Cells

The nuclear factor of activated T cells (NFAT) was previously identified as an inducible nuclear factor binding to the promoter of the interleukin-2 (IL-2) in activated T-cells (Shaw et al., 1988). Gradually, NFAT has been found to have a regulatory role in other organs including the central nervous system, blood vessels, heart, kidney, bone skeletal muscle, and HSCs (Macian, 2005). It is regulated by Ca2^+^ and the Ca2^+^/calmodulin-dependent serine phosphatase calcineurin leading to a direct link between the intracellular Ca2^+^ signaling and gene expression. Upon stimulation, calcineurin-dependent dephosphorylation of NFAT proteins steers them for nuclear translocation followed by transcription activation (Hogan et al., 2003). Balanced signaling of the Ca/NFAT pathway in the hematopoietic stem and progenitor cells are extremely necessary for the developing embryo. The importance of the NFAT pathway has been demonstrated by generating transgenic mice having hyperactive NFAT1 or increased Ca/NFAT signaling or reduced phosphorylation of the NFAT1 by decreasing the affinity of the kinase due to changes in the docking site of NFAT. In these experiments, it was found that hyperactive NFAT1 had deleterious effects during the early embryonic development and also reduced the number of terminally differentiated B and T lymphocytes from HSCs (Müller et al., 2009). Ca/NFAT signaling pathway is of utmost importance for the development and differentiation of the T lymphocytes from the thymocytes in the thymus (Macian, 2005). In Nfat deficient mice, a reduction in the number of single-positive thymocytes has been observed which correlates with increased apoptosis of the double-positive thymocytes (Oukka et al., 1998). Calcineurin-NFATc3 binds to the promoter of Etv2 to regulate its transcription, which is a key factor for hematopoiesis. The inositol 1,4,5- triphosphate receptors (IP3Rs) Ca2^+^ signaling pathway that is another mediator of the Calcium-calcineurin-NFAT pathway, is also essential for the early development of the embryo. The deletion of IP3Rs resulted in reduced hematopoietic mesoderm, hematopoietic progenitor cell population, and the colony-forming unit activity. The IP3R knockout mice also exhibited reduced Etv2 expression which in turn also resulted in the reduction of the hematopoietic development (Wang et al., 2017). NFAT in the osteoblasts regulates the expression of vascular cell adhesion molecule 1 (VCAM-1) on the osteoblasts, a mediator of the cell adhesion and signaling during leukocyte development. A mouse model with a dominant-negative Nfat also had decreased production of the B-cell lineage through the reduction of the VCAM-1 (Sesler and Zayzafoon, 2013). Detailed studies have revealed that signaling through Ca2^+^/calcineurin/Nfatc3, c4 in the tissues surrounding vessels during early embryonic development prevents aberrant patterning of the vasculature and mediates proper anatomical patterning of the vascular system between E7.5 and E8.5 (Graef et al., 2001). Calcineurin/Nfat participates in the formation of the vertebrate heart valve in an evolutionarily conserved manner from zebrafish to the mammals. It functions sequentially from the myocardium to the endocardium within a valvular morphogenetic field to commence and continue the embryonic valve formation. Studies unveil that initiation of the morphogenesis of the heart valve requires the calcineurin/Nfat signaling to repress the VEGF expression in the myocardium underlying the site of future valve formation. This repression of VEGF at E9 is important for the endocardial cells to transform into mesenchymal cells. Again, at E11, the second wave of calcineurin/Nfat signaling is needed in the endocardium, neighboring to the earlier site of action in the myocardium to direct the elongation and fine-tune the valve formation. Thus, calcineurin/NFAT signaling is indispensable for heart valve formation (Chang et al., 2004). It also plays an essential role in cardiac muscle development. Nfatc3 and Nfatc4 combinatorial gene disrupted embryos resulted in abnormal heart morphogenesis by E10.25 and embryonic lethality at E11. Cellular, molecular, and metabolic analysis in these knockout mice reveals decreased cardiomyocyte proliferation and compromised cardiac mitochondrial architecture and function at E10.5 (Bushdid et al., 2003). Therefore, it is very evident that Nfatc3/4 is required for the maintenance of cardiomyocyte functions and bioenergetics during development. It is required temporally for the development of the atrial myocardium and regulates transcription of cardiac troponin genes, therefore playing a critical role in the structural architecture of the developing myocardium (Schubert et al., 2003). Nfatc1 is an important determinant of the lymphatic EC patterning in the developmental as well as injury-induced lymphangiogenesis. In Nfatc1 null mice, the ECs after sprouting and migrating away from the cardinal vein coalesced poorly forming smaller lymph sacs (Kulkarni et al., 2009). Its signaling is also crucial for coronary artery angiogenesis within the epicardium by activating Smad2 and TGFβ-Alk5 signaling pathways (Zeini et al., 2009). A co-dependent signaling module in the cardiomyocytes involving the calcineurin/NFAT and MEK1-ERK1/2 pathways regulates the cellular growth response which consequently leads to hypertrophy (Sanna et al., 2005). Deletion of Nfatc4 did not compromise the ability of the myocardium to undergo hypertrophic response but the loss of Nfatc3 resulted in the partial reduction of calcineurin-transgene induced cardiac hypertrophy in mice (Wilkins et al., 2002). This data suggests that NFATc3 is regulated downstream after calcineurin activation and plays a more pivotal role in causing hypertrophy than other NFATc factors. NFATc1 is involved in the motility of VSMCs in response to the receptor tyrosine kinase (RTK) and G-protein coupled receptor (GPCR) agonist, therefore, having a prominent role in vascular wall remodeling (Liu et al., 2004). Angiotensin II activated calcineurin/NFATc3 signaling leads to arterial dysfunction and subsequent hypertension due to the downregulation of the β1 subunit of the Ca2^+^-activated K^+^ channels (Nieves-Cintrón et al., 2007). NFATc1 is found to be a major regulator of restenosis by targeting cyclinA/CDK2 during VSMC multiplication (Karpurapu et al., 2008). The NFAT family is one of the major transcription factors involved in the development of the embryo from the hematopoietic development to the vascular patterning and also takes part prominently in the causation of the cardiac diseases. NFATc1 has been implicated in the formation of neointima as well by increasing the cox-2 levels in the arterial walls (Liu et al., 2005). It is also involved in the regulation of pulmonary arterial hypertension by getting activated in the inflammatory cells as well as in the pulmonary arterial smooth muscle cells (PASMCs) (Chen et al., 2017). Therefore, NFATs’ are highly involved in the hematopoietic and vascular wall formation during the early development and also in the initiation of arterial diseases in the later stages of life.

### LIM Domain Only Protein – 2 (LMO2)

The **L**I**M** domain **O**nly protein – 2 (LMO2) was first discovered as the recurrent chromosomal translocation partner of a TCR locus in a subset of patients with T-cell acute lymphoblastic leukemia (T-ALL) (Chambers and Rabbitts, 2015). LMO2 formerly known as RBTN2, Rhom-2, or Ttg-2, comprises the **LIM**-domain which includes the transcription factors **L**in-11, **I**sl-1, and **M**ec-3. These are cysteine-rich motifs with consensus amino acid sequences (Chambers and Rabbitts, 2015). Although this gene was first reported as an oncogene, later, it was found to be very important in fetal development and exclusively expressed in the nucleus of the human hematopoietic cells (Neale et al., 1995). LMO2 plays an important role in the formation of the oligomeric complex that binds to the DNA during hematopoiesis and particularly erythroid development. In the erythroid cells, it forms the oligomeric complex with GATA-1, SCL, E2A, and LIM binding protein Ldb1/NL1 which binds to a unique bipartite DNA motif known as E-box (Wadman et al., 1997, GATA-1 and Ldb1/NLI proteins the GATA-1 gene in embryonal stem (ES) cells, which are The phenotypes of the). Overexpression of LMO2 leads to the inhibition of hematopoiesis by having dominant-negative inhibitory effects on erythropoietic development. Mutation in Lmo2 results in the death of the fetus, miniature liver, and heart formation, decreased hematopoiesis, and hypoplastic thymus (Terano et al., 2005). LMO2 is regulated post-transcriptionally as well as post-translationally in the erythroid progenitors to maintain the fidelity of erythroid differentiation (Brandt and Koury, 2009). The shRNA-mediated knockdown of Lmo2 in primary erythroblasts led to the observation that it regulates prototypical erythroid genes (Brandt and Koury, 2009). Reducing its level in the erythroid progenitors hinders G1-S progression and ceases erythropoietin-dependent cell growth while supporting terminal differentiation. On the other hand, ectopic expression of Lmo2 in the thymocytes induces DNA replication while blocking the differentiation (Sincennes et al., 2016). Embryonic stem cells of the Lmo2^–/–^ mouse differentiated less efficiently into hemogenic endothelium which failed to generate definitive hematopoietic progenitors but inefficiently promoted primitive hematopoietic progenitors (Sincennes et al., 2016). Transgenic zebrafish expressing either EGFP or DsRed under the *lmo2* promoter was sufficient enough to generate blood and vascular development. Transplantation of Lmo2^+^ cells from these transgenic embryos during primitive hematopoiesis exhibits that these cells could generate blood and blood vessel cells in the recipients. A critical *cis*-element containing functional ETS-domain transcription factor binding sites (EBS) was identified which implies ETS factors as regulators of *lmo2* during early hematopoietic and vascular development (Zhu et al., 2005).

Although LMO2 does not take part in cardiac development, its role is essential during early mesodermal specification and endothelial differentiation and angiogenesis. Lmo2 null embryonic stem cells are unable to commit to the vascular ECs after E10.5 (Yamada et al., 2000). Similar to hematopoiesis, LMO2 along with its associated factors forms a DNA-binding complex that controls the target gene expression required for angiogenesis (Yamada et al., 2000). The ‘loss of function’ studies of LMO2 along with its binding partner SCL has indicated that these factors play a major role in hematopoiesis and angiogenic remodeling of the vasculature; also, the ‘gain-of-function’ studies manifest that these factors have a role early in the specification of the hemangioblasts which are the putative bipotential precursors of blood and endothelium (Gering et al., 2003). SCL acts synergistically with LMO2 in the early mesoderm and ectopically induce the expression of its binding partner LMO2 in the hemangioblasts like cells in the tissue. Furthermore, in the absence of the inducers of the erythroid and myeloid development, the Scl/Tal1-Lmo2-induced hemangioblasts differentiate into ECs (Gering et al., 2003). Additionally, identification of a novel interaction of LMO2 with Ets factors imparts support to the fact that LMO2 is the indispensable factor for the emerging regulatory network that controls mesodermal differentiation toward hemangioblasts and subsequently to blood and endothelial lineage (Landry et al., 2005). In zebrafish, Lmo2 is found to be a critical determinant of angiogenesis and vascular regeneration, suggesting that role of Lmo2 is conserved across the species (Meng et al., 2016). Rather than the involvement of Lmo2 in the causation of CVDs, it mostly contributes toward tumor angiogenesis resulting in the nourishment and spread of the tumor and therefore serves as an anti-angiogenic drug target (Yamada et al., 2002).

### Forkhead Box Family

The Forkhead box or the FOX family of transcription factors in mammals share homology with the forkhead gene found in *Drosophila* hence suggesting a critical evolutionarily conserved biological role (Cabrera-Ortega et al., 2017). All the members of these FOX families share a 110-amino acid DNA-binding domain known as the “forkhead box” or “winged helix” domain (Tothova and Gilliland, 2007). The FOX family of transcription factors are the largest family of transcription factors known in humans and have been grouped into 19 subfamilies (FOXA-FOXS) according to their sequence similarity (Tothova and Gilliland, 2007). Among these, some of the subfamilies, along with their subgroups are involved in different ways with the maintenance of the HSCs as well as the vascular progenitor cells. Foxc1 is one of the essential transcription regulators for the maintenance of the mesenchymal niches for the HSCs and progenitor cells (Omatsu et al., 2014). In Foxc1 deleted mice, there was a reduction in the development of the HSCs and Hematopoietic Progenitor cells (HPCs) as mesenchymal niches were replaced by adipocytes (Omatsu et al., 2014). Foxm1, another subfamily of the Fox family of transcription factors, acts as a key regulator in all transition phases of the cell cycle stages (Hou et al., 2015). Hou et al. (2015) have proved that loss or reduction of Foxm1 in both mice and humans reduces the frequency of the quiescent HSCs and increases the proliferation of the HSCs and the HPCs although not affecting their differentiation. Loss of Foxm1 induces downregulation of the cyclin-dependent kinase inhibitors which includes p21 and p27 leading to the direct suppression of the gene encoding Nurr1(Nr4a2), which is a critical regulator of the HSC quiescence. Thus, through the Nurr1 mediated pathway, Foxm1 regulates the quiescence and self-renewal capacity of the HSCs (Hou et al., 2015). HSCs mostly remain in the quiescent stage to preserve the self-renewal capacity and consequently enable the life-long hematopoiesis. The FOXO subfamily is subdivided into FOXO1, FOXO3, FOXO4, and FOXO6, of which FOXO1, FOXO3, and FOXO4 that are ubiquitously present and play an essential role during the developmental stages. In Foxo3a^–/–^ mice self-renewal capacity of HSCs is completely lost due to the defects in the quiescence maintenance (Miyamoto et al., 2007). Among these genes, FOXO3 is of utmost importance for the maintenance of the homeostasis and regulation of oxidative stress in the HSCs (Yalcin et al., 2008). Foxo3^–/–^ mice have functionally defective HSCs mediated by ineffective regulation of oxidative stress. The results also stated decreased quiescence and impaired G2/M transition in Foxo3 deficient HSCs. Also, FOXO3 is required for the ataxia telangiectasia mutated (ATM) gene expression which is crucial for the HSC self-renewal (Yalcin et al., 2008). It is also critical for the regulation of the respiration carried by mitochondria in the HSCs (Rimmelé et al., 2015). Mitochondrial dysfunction is one of the main causes of the higher ROS levels in Foxo3^–/–^ HSCs.

Additionally, Foxc1 is a very important transcription factor for vascular development as well. Foxc1 is essential for the differentiation of the VSMCs and vascular basement membrane integrity in Zebrafish (Skarie and Link, 2009; Whitesell et al., 2019). Foxc1 and Foxc2 compound mutants have shown three major defects in early cardiovascular development which are – a failure in the remodeling of the primitive vascular plexus of the head, trunk, and yolk sac into a branched system of large and small blood vessels; defective development of the heart which is smaller in size than the normal without undergoing complete morphogenesis; and abnormality in the number, size, and organization of the branchial arteries that leads to their lethality (Kume et al., 2001). Both Foxc1 and Foxc2 cooperatively control the sprouting of the lymphatic ECs and critically control the proper process of the arterial specification (Seo et al., 2006). A novel mutation in the FOXC1 gene was reported in a three-generation family with three Axenfeld-Rieger Syndrome (ARS), a developmental disorder affecting structures in the anterior segment of the eye and heart defect patients. This fact identifies the role of FOXC1 in Congenital Heart Disease (CHD) and ARS. This particular mutation is located at the forkhead DNA binding domain of the FOXC1 gene which is highly conserved for all the forkhead transcription factors and binds to the conserved DNA sequences of the target genes (Du et al., 2016). Dysregulation of FOXC1 or its downregulated gene leads to CHDs including coarctation of the aorta as well (Sanchez-Castro et al., 2016). Lineage tracing analyses manifests that Foxc2 expressing neural crest cells are involved in the development of aorta, pulmonary trunk, valves, and endocardial cushions. Foxc2^–/–^ embryos died between E12.5 andE13.0 as every embryo had persistent truncus arteriosus and ventricle hypoplasia. The following fate map and gene expression analyses stipulated that Foxc2 played a very critical role in regulating the proper migration of the neural crest cells, outflow tract separation, and ventricle development (Inman et al., 2018).

Foxm1 induces TGF-β and plays a critical role in the development of cardiac fibrosis through the endothelial-to-mesenchymal transition (EndMT) which is another form of epithelial-mesenchymal transition (EMT) (Song et al., 2019). Increased activation of FoxO3 led to increased autophagy, subsequently playing a protective role in the cerebral ischemic reperfusion injury in penumbral rat brain tissue (Zhou et al., 2019). FoxO factors take part in the cell growth of the heart and therefore participate in hypertrophic conditions. Inhibition of the calcineurin/NFAT signaling cascade by FoxO and eventually rescuing the repression by PI3k/Akt pathway are important mechanisms by which FoxO factors govern the cell growth in the heart (Ni et al., 2006). FoxOs are important regulators of cardiac disease and have been implicated in ischemic cardiac diseases (Xin et al., 2017).

### Runx Family

The Runt box transcription factor (Runx) belongs to the family of the core-binding factor (CBF). Runx1, Runx2, Runx3 are well conserved in mammals that encode proteins involved in maintaining the blood cell lineages and vascular development. The CBF alpha subunit of the Runx interacts with the CBF beta subunit to form a complex which binds to the DNA and regulates critical cellular processes. Several animal studies have illustrated the central role of Runx in the hematopoietic development and any mutation in this transcription factor has exhibited complete loss of hematopoietic lineage commitment (Wang et al., 1996; Swiers et al., 2010). Runx interacts with other transcription factors like GATA1, Ets1, Pu.1, Myb and they work synergistically in regulating the activation of target genes.

Runx1 is mainly responsible for the differentiation and self-renewal of HSCs whereas Runx2 and Runx3 are involved in the maintenance of HSCs (de Bruijn and Dzierzak, 2017). The expression of Runx1 has been ascertained very early during the developmental process starting from E7.5 in the mesodermal layer. Runx1 knockout results in embryonic lethality between E11.5–E12.5 due to extensive hemorrhages (Wang et al., 1996). Runx-Cre mouse models have been used to understand the early Runx expression pattern in the hematopoietic sites and cells. Haploinsufficiency leads to the irregular spatiotemporal distribution of HSCs in mice with the early appearance of HSCs in the yolk sac and aorta. These mice also show premature differentiation of hemangioblasts and mesodermal development. However, the conditional deletion of Runx1 has minimal effects on the number and phenotype of long-term repopulating HSCs (Cai et al., 2011; Mevel et al., 2019). Loss of functional Runx1 perturbs the lineage-specific maturation of B-T cells and also inhibits the production of common lymphoid progenitors. MX-Cre mediated deletion of Runx3 in aged mice exhibited myeloproliferative disorder and increases the frequency of hematopoietic progenitor cells (Sherwin and Sacca, 1984; Chen et al., 2009). Double knockout of Runx1/3 leads to embryonic death within 25 weeks due to bone marrow failure and excessive myeloproliferative phenotype (Wang et al., 2014). This signifies the regulatory role of Runx in adult hematopoiesis and myeloproliferative disorder.

Runx1 is well known for its involvement in embryonic heart development. Runx1 knockout mice have shown several phenotypic changes such as ventricular septal defect, thin myocardium development, and underdeveloped coronary plexus, which reveal its importance during heart development (Lluri et al., 2015). Besides, Runx1 mediates the transition of hemogenic endothelium to hematopoietic cells. This transition is blocked in murine models with the absence of Runx1 expression in the yolk sac that causes embryonic lethality within the mid-gestation period (North et al., 1999; Riddell et al., 2020). Similar transitional dysfunctions were observed in zebrafish and chick embryo, where blocking the activity of Runx1 shows the complete absence of hematopoietic cell cluster and functional hematopoietic progenitor cells (Tavares et al., 2018). Although the expression of RUNX1 is high during the neonatal period and subsequently decreases in the adult heart, studies have reported a hike in its expression in the course of cardiac pathology. In patients with an ischemic heart condition, an acceleration in the expression levels of 52 kDa isoform of RUNX1 was observed compared to the control samples (Levanon et al., 2001). The upregulation of the Runx1 expression has been observed under several stress conditions such as pressure overload, dilated cardiomyopathy, and diabetic cardiomyopathy. Several clinical and animal studies support the overexpression of RUNX1 in the heart during cardiovascular pathology (Gattenlöhner et al., 2003; Lluri et al., 2015; McCarroll et al., 2018). In a recent study, the upregulation of Runx1 was observed in cryo-injured heart tissues. This surge in Runx1 blocks the expression of cardiac repair genes in cardiomyocytes, myofibroblast, and ECs where they inhibit the genes involved in cardiac repair (Koth et al., 2020). All these studies highlight the importance of Runx in maintaining definitive hematopoiesis and in the onset of hematological dysfunctions. Moreover, these studies indicate a major role of Runx1 in the heart and vascular development and also under cardiac dysfunction specifies them as emerging therapeutic targets.

### ETS Family

ETS (E twenty-six) transcription factors constitute a large evolutionarily conserved gene family (Sharrocks et al., 1997). These factors are characterized by sequence homology in their DNA binding ETS domain. The ETS domain forms a winged helix-turn-helix structural motif, that binds to a consensus sequence in the DNA, GGAA/T, found in the target genes (Sharrocks, 2001). The name of this protein family originated from the name of the avian erythroblastosis virus, E26, that carried *v-ets* (E twenty-six) oncogene (Sharrocks, 2001). Although the ETS family of transcription factors share the conserved ETS binding domain, yet the sequence divergence neighboring the ETS domain as well as the differences in the amino-acid sequences have a profound effect on the individuality and functionality of the protein members of this family (Sharrocks, 2001). Several proteins of the ETS family have been shown to play crucial roles in the hematopoiesis, including PU.1, FLI1, and TEL/ETV6 (Lacadie and Zon, 2011). PU.1 is indispensable in hematopoietic development (Oikawa et al., 1999). It is highly expressed in most of the cells of the hematopoietic lineage – erythroid, monocytic, granulocytic, and B-lymphoid lineages (Hromas et al., 1993; Pettersson et al., 1995; Zhang et al., 1996). Embryos carrying homozygous mutations in the PU.1 gene did not develop beyond late gestational stage and showed impaired erythroblast maturation (Scott et al., 1994). Also, multilineage defects in the generation of the progenitors for B and T lymphocytes, monocytes, and granulocytes were observed as an invariant consequence (Scott et al., 1994). Mice homozygous for the disruption of the PU.1 DNA binding domain were born alive but died of severe septicemia within 48 h. Analysis of these neonates unveiled a lack of mature macrophages, neutrophils, B cells, and T cells, although erythrocytes and megakaryocytes were present (McKercher et al., 1996). Genetic manipulation studies in mice revealed that *Fli-1* is involved in multiple roles in the hematopoietic development along with the development of the megakaryocytes and the platelets (Kruse et al., 2009). Fli-1 and another member of the ETS family, Erg, share greater homology among each other than to other members of the ETS family and are indispensable in the hematopoiesis (Kruse et al., 2009). Mice with homozygous mutations in the Erg gene died between E10.5 and E13.5 due to defective definitive hematopoiesis and they are well known for their involvement in adult hematopoiesis (Kruse et al., 2009; Lacadie and Zon, 2011). ERG is critical for HSC self-renewal and regulates megakaryopoiesis, angiogenesis, endothelial apoptosis along with its requirement in endothelial fate by ESC differentiation (Lacadie and Zon, 2011). During the embryonic development, Ets1 levels are higher in the blood islands of the yolk sac, where the hemangioblasts, the common precursors of hematopoietic and vascular lineages are located (Pardanaud and Dieterlen-Lièvre, 1993). A unique member of the ETS family is the GA binding protein (GABP). It is the only obligate multimer, that forms an active tetramer by assembling a complex with GABPα and β (Yang et al., 2013). The deletion of Gabpα gene in the bone marrow arrests the cell cycle in the HSCs (Yang et al., 2013). Other members of ETS such as Elf1, TEL1, SpiB are pivotal in the differentiation of lymphoid lineage and notably expressed in the hematopoietic tissue of adult mouse and human (Su et al., 1996, 1997; Wang et al., 1998; Maroulakou and Bowe, 2000). ETS transcription factors play a crucial role in the hematopoietic development and simultaneously they are well known for their involvement in embryonic heart and vascular development.

Among all the ETS genes, about thirteen members of the family are expressed in the hematopoietic or ECs and are thus involved in the hematovascular development in the early vertebrate embryogenesis (Craig and Sumanas, 2016). Among these, ETV2 or Er71 is extremely crucial in both hematopoietic as well as endothelium development (Lee et al., 2011; Sumanas and Choi, 2016). This protein also contributes to the maintenance of the adult HSCs and regulates its normal functionality (Lee et al., 2011). The ablation of the ETV2 gene in the mice leads to embryonic death in early gestation due to fewer hematopoietic cells and lack of vascular structures (Lee et al., 2011). ETV2 has also been crucial in specifying the endocardial fate and transiently expressed in the endocardium and endothelium of the developing embryo (Lee et al., 2011). Deficiency of ETV2 results in the complete block of the blood and endothelial development followed by embryonic lethality, indicating the indispensable role of ETV2 during hematopoiesis and vessel development (Liu et al., 2015). Besides, ETV2 directly activates other ETS genes therefore, establishing an ETS hierarchy that maintains the blood and EC development (Liu et al., 2015). These two genes are associated with cardiac cushion development in the second heart field (Bosman et al., 2015). ETV6/TEL controls the optimal threshold of the inhibitors of angiogenesis and is responsible for priming the ECs for sprouting (Roukens et al., 2010). Fli1 is involved in the maintenance of vascular integrity and is essential for EC viability, while Erg is responsible for the maintenance of vascular integrity, angiogenesis, EC development, and blood vessel maintenance (Craig and Sumanas, 2016). Both Ets1 and Ets2 are involved in regulating the factors responsible for angiogenesis, and Ets1^–/–^Ets2^–/–^ double mutants exhibit defects in angiogenesis and cell apoptosis, whereas individual mutants do not show any vascular phenotypes (Wei et al., 2009).

Overexpression of ETS2 and ERG, located on chromosome 21 in humans is responsible for the congenital heart disease (CHD) in Down Syndrome patients (Bosman et al., 2015). The Ets-2 expression is decreased in the circulating Endothelial progenitor cells (EPCs) in patients with advanced stages of CVDs (Tejerina et al., 2017). This lower expression of Ets-2 in these cells, explains the poor functionality of the EPCs in hyperlipidemia or patients undergoing coronary artery bypass grafting (Tejerina et al., 2017). ETS transcription factors have been implicated in the regulation of vessel-specific genes in vascular inflammation as well as in remodeling. Mostly, the ablation of the Ets genes results in the defects of the immune cells, which in turn also causes inflammation in the vessels modulating the inflammatory responses (Oettgen, 2006). ETS family is mostly involved in the wholesome development of the embryo compared to its potential in causing CVDs.

## Epigenetic Modifications of Transcription Factors

Epigenetic modification primarily concerns with histone modification that shapes the HSC landscape from the very beginning in embryonic development as well as during maintenance and diseases. Proteins that package the DNA into nucleosomes are known as the histone proteins. Enzymes such as histone methyltransferases (HMTs), histone demethylases (HDMs), histone acetylases (HATs), histone deacetylases (HDACs), etc. are involved in modifying key residues of histones that ultimately leads to spatial and temporal activation and suppression of genes in stem cells. The N-terminal tail of the histone subunits that protrude from the nucleosome core predominantly undergoes these modifications by the aforementioned enzymes. These enzymes are grouped into different families responsible for the modifications of the chromatin structure and gene accessibility. Additionally, the DNA undergoes another principal epigenetic modification which is the methylation of the C5 position of the cytosine residues in the CpG dinucleotide sequences. For instance, de novo DNA methylation and maintenance of these CpG islands are executed by a family of HMTs known as DNA methyltransferases (DNMTs). Furthermore, DNA methylation plays a crucial role in HSC self-renewal, maintenance of the stem cell pool, and tightly controlling the differentiation into different cell populations. Any aberrant change in the DNA methylation impairs the differentiation process thus leading to disproportionate proliferation. Almost all of these signatures are erased and rewritten and affect the post-fertilization phenotype. These modifiers in association with transcription factors and other cofactors create an intricate regulatory network that modulates HSC development, maintenance, and differentiation, as well as cardiogenesis and CVD progression (Mizuno et al., 2001; Young et al., 2004; Huo and Zhang, 2005; Rice et al., 2007; Bröske et al., 2009; Challen et al., 2011; Elizalde et al., 2012; Webster et al., 2013; Broxmeyer, 2014; Liao et al., 2015; Sharma and Gurudutta, 2016; Zhang X. et al., 2016).

### In Hematopoietic Stem Cells

The role of transcription factors in the modulation of hematopoiesis has been well studied in the last three decades (Kehrl, 1995; Zhu and Emerson, 2002; Vicente et al., 2012). Here we discuss the interaction of epigenetic modifiers such as HDMs, HDACs, HATs, and HMTs with transcription factors in hematopoiesis modulation. DNA methylation plays a crucial role in HSC self-renewal and maintenance of the stem cell pool as well as tightly controlling the differentiation into different cell populations. Studies in Dnmt1 deficient mice have shown that strong methylation of GATA1 promoter region is essential for maintaining the “stemness” in HSCs. Dnmt1 deficiency induces the hypomethylation of the GATA1 gene in HSC causes disproportionate differentiation to myeloerythroid lineage (Bröske et al., 2009; Trowbridge et al., 2009). Similarly, histone methylation has been shown to modulate the activity of transcription factors in HSCs. Mice deficient in an HMT called Disruptor of telomere silencing 1-like (DOT1L) has been shown to have significantly reduced GATA-2 in their embryonic stem cells (Feng et al., 2010). Similarly, Mixed Lineage Leukemia (MLL) which also falls under the category of the HMTs, forms complex with RUNX1. This complex of RUNX1 and MLL is required to maintain the trimethylated mark on the lysine 4 of H3 histone (H3K4me3) at two crucial regions of the RUNX1 target gene PU.1 (Huang et al., 2011; Koh et al., 2013).

On the other hand, HDMs like Lysine-specific demethylase 1 (LSD1) removes methyl groups from the 4th lysine (K4) of the N-terminal tail of H3 histone, hence also known as H3K4 demethylase. LSD1 has been shown to repress the transcription activity of GATA-2 during hematopoiesis and drive the differentiation toward erythroid lineage (Guo et al., 2015). Binding of LSD1 to SCL regulates HSC differentiation and hematopoiesis *in vitro*. Any mutation or genetic deletion of LSD1 resulted in impaired myeloid cell lineage development (Hu et al., 2009). Another HDM like KDM3B has been shown to turn on the transcription activity of transcription factors like LMO-2 (Kim et al., 2012). The effect of epigenetic modifiers on the transcription activity varies with each interaction contributing to the complex regulatory network. HATs such as CBP and P300 acetylate GATA-1 regulating the process of HSC renewal (Boyes et al., 1998; Rebel et al., 2002). Additionally, P300 and GCN5 acetylate GATA-2 and regulate its DNA binding activity (Hayakawa et al., 2004). HDACs like HDAC-4 and HDAC-5 deacetylate GATA-1 and repress its transcription activity (Watamoto et al., 2003). HDAC3 and HDAC5 have been found to co-localize and interact with GATA-2 in HSCs. HDAC3 significantly repress GATA-2 transactivation potential (Ozawa et al., 2001). The interplay among HATs, HDACs, and transcription factors like GATA-1, GATA-2, NF-E2, EKLF, TAL-1/SCL, C-myb, and PU.1 plays a crucial role in the modulation of hematopoiesis. For instance, PU.1, an ETS family member can inhibit the activity of HATs like CREB-binding protein (CBP), a HAT to alter expression levels of other hematopoietic transcription factors such as GATA-1, EKLF, NF-E2 as well as its expression can be altered the by HDAC inhibition (Laribee and Klemsz, 2001; Hong et al., 2002; Huo and Zhang, 2005; Gupta et al., 2006).

In induced pluripotent stem cells, another HDAC, SIRT2 (NAD-dependent deacetylase sirtuin 2) has been shown to modulate hematopoiesis by deacetylation of LMO-2 and initiates differentiation toward myeloid lineage (Morishima et al., 2015, 2019). SCL interacts with co-activators like p300 and co-repressors like msin3A to regulate the hematopoiesis and erythroid lineage commitment during differentiation. HATs like p300/CBP associated factor (P/CAF) acetylates SCL. This acetylation of SCL leads to enhanced DNA binding activity of SCL and also activates the genes involved in erythroleukemia cell differentiation (Huang et al., 2000). mSin3A along with HDAC1 binds to SCL and limits the differentiation of erythroid cells (Huang and Brandt, 2000). The ability of HDACs like Mta3-NuRD has been shown to affect the activity of crucial hematopoietic transcription factors like SCL, LMO2, GATA-1, and GATA-2 during primitive hematopoiesis in Zebrafish embryo (Li et al., 2009). The interplay among HATs, HDACs and transcription factors like GATA-1, GATA-2, NF-E2, EKLF, TAL-1/SCL, C-myb, and PU.1 plays a crucial role in the modulation of hematopoiesis. For instance, PU.1, an ETS family member can inhibit the activity of HATs like CBP, a HAT to alter expression levels of other hematopoietic transcription factors such as GATA-1, EKLF, NF-E2 as well as its expression can be altered the by HDAC inhibition (Laribee and Klemsz, 2001; Hong et al., 2002; Huo and Zhang, 2005; Gupta et al., 2006). In some cases, multiple epigenetic modifiers work synergistically to affect the transcription activities of several proteins. KDM3B, an HDM forms a coactivator complex with CBP, which is a HAT and enhances the transactivation of LMO-2 (Kim et al., 2012). Another instance of synergistic effect is that RUNX1 forms a corepressor complex with HDACs and HMTs. Sin3a, another HDAC1, and HMTs like protein arginine methyltransferase 6 (PRMT6) impart asymmetric histone H3 arginine-2 dimethylation (H3R2me2a) at megakaryocytic genes in human hematopoietic progenitor cells. This event maintains the progenitor cells in an intermediate state in which the differentiation genes remain in a suppressed state but poised for rapid transcription activation (Herglotz et al., 2013).

### In Cardiovascular Pathology

A similar interactive network between the cardiac transcription factors and epigenetic modifiers also maintains the cardiovascular lineage commitment of cells during cardiogenesis. Several studies in recent years have focused on the role of epigenetic modifications in cardiac progenitor cell differentiation and aberrations in epigenetic changes leading to CVDs (Handy et al., 2011; Zhou et al., 2011; Chamberlain et al., 2014; Arcidiacono et al., 2018). Epigenetic modifiers like HMTs and HATs play a crucial role during the differentiation process due to their interaction with several cardiac-specific transcription factors like Nkx2.5, Mef2c, Gata4, Tbx5. These interactions result mostly in promoter region modifications and thus further regulate the expression of several downstream cardiac-specific genes (Liu et al., 2009; Coppola et al., 2014; Burridge et al., 2015). Several histone modifiers have been shown to partner with Mef2c and regulate cardiac-specific differentiation. Class IIa HDACs, which remove lysine acetylation form a complex with Mef2c resulting in a “closed” chromatin conformation and thus lead to transcription repression (Zhou et al., 2011). In the cardiac hypertrophic pathway, MEF2 is well studied as a downstream target of HDAC9. In a study involving HDAC9 mutant mice, MEF2 was found to be “super-activated” and interacting with NFAT and GATA transcription factors. This further suggests that HDACs possess the potential to repress hypertrophy responsive genes via. Mef2 (Zhang et al., 2002). Forkhead box transcription factors like FOXO interact with SIRT1 (silent mating type information regulation 2 homologs 1), which is a member of the sirtuin family of the class III histone deacetylases. Activation of SIRT1/FOXO mediated anti-oxidative stress response protects against ischemic reperfusion injury in CD38 deficient mice (Guan et al., 2016). Similarly, ETS family members like ets-1 and ets-2 have been shown to interact with HATs like p300 and CBP to upregulate matrix metalloprotease 3 (mmp3), a gene implicated in CVDs (Jayaraman et al., 1999; Creemers et al., 2001; Beyzade et al., 2003; Pons et al., 2009). This shows that the interactive network of transcription factor with HATs and HDACs plays an important role in vascular remodeling and CVDs and can be potential therapeutic targets. Other epigenetic modifications by HMTs have shown to be involved in cardiac differentiation. SET, MYND domain-containing 1 (Smyd1), an important H3K4 methyltransferase has been shown to bind downstream of Mef2c and regulate the cardiac differentiation and morphogenesis. The deletion of Smyd1 in mice causes lethality during the early embryonic developmental stages due to dysregulation in cardiac cell maturation (Gottlieb et al., 2002). DNA methylation has been shown to affect the activity of transcription factors involved in the differentiation of cardiac progenitor cells into mature cardiomyocytes. Additionally, DNA methylation along with histone acetylation modulates the transcription activity of GATA4 and drives the differentiation of mesenchymal stem cells (MSCs) to cardiomyocytes like cells (Aibin and William, 2012; He et al., 2012; Jiang et al., 2017). PRC2, a DNA methyltransferase has recently been shown to repress the transcription activity of GATA4 by direct methylation, depositing H3K27me3 epigenetic marks in both *in vitro* and fetal hearts. Mef2c plays an important role in cardiac reprogramming by regulating the expression of several cardiac remodeling factors. Overexpression of Mef2c along with GATA4 and Tbx5 can cause direct reprogramming of cardiac fibroblast to myocytes (Ieda et al., 2010). Thus, the interactions of epigenetic modifiers with Mef2c is of biological significance, specifically to understand cardiomyogenesis.

Histone acetylases interact with transcription factors and activate the expression of cardiac-specific genes. p300 an important HAT has been shown to activate the complex of Mef2c and GATA4 by increasing their DNA binding ability. This complex plays a significant role in driving the differentiation process of embryonic stem cells toward cardiomyocytes (Kawamura et al., 2005; Zheng et al., 2013; Yilbas et al., 2014). Besides, a study using p300 knockout mice reported reduced trabeculation during the early heart development resulting in embryonic lethality at E9.0-11.0 (Yao et al., 1998).

Despite the growing data contributing to the understanding of the interactions in the intricate network of transcription factors and epigenetic modifiers, there remain gaps in our knowledge. This demands further research into the targets of the epigenetic modifiers as well as the underlying mechanisms of the epigenetic modification processes themselves. Further research in this area will provide us with an opportunity to fully comprehend and design therapeutic strategies to treat CVDs.

## Conditional Differentiation of HSC During CVDs

The underlying pathophysiology of CVDs is most often oxidative stress/inflammatory (chronic) stress/mechanical stress-induced damage to cardiomyocytes, VSMCs, and ECs. These cardiovascular pathological conditions cause the recruitment of immune cells which secrete not only growth factors and pro-inflammatory cytokines but also promote adverse remodeling of the heart and vessels. This cardiac and vascular immune component governs the phenotypic switching and proliferation of the smooth muscle cells (SMCs) and endothelial dysfunction leading to a pathological condition associated with damage to the vascular wall. VSMCs’ phenotypic switching and proliferation along with endothelial dysfunctions are the major driving forces behind the pathogenesis of vascular remodeling during atherosclerosis and restenosis. VSMCs change from a “quiescent and contractile” to a “synthetic and proliferative” phenotype, thereby causing neointimal hyperplasia. It is believed that these cells behave partly like stem cells and secrete extracellular matrix proteins, consequently contributing to the narrowed lumen and stenosis (Higashi et al., 2009; Orlandi and Bennett, 2010; Lim and Park, 2014). Several studies have indicated that these proliferative VSMCs might have different sources of origin. These sources include HSCs as well as multipotent progenitor cells, MSCs, circulating smooth muscle progenitor cells, resident smooth muscle progenitor cells within blood vessels, and even multipotent cells from other tissues like adipose tissue in both humans and mice (Reyes et al., 2002; Hirschi and Majesky, 2004; Hu et al., 2004; Matthews et al., 2006; Rodríguez et al., 2006; Sugiyama et al., 2006; Jiang et al., 2007; Tanaka et al., 2007; Orlandi and Bennett, 2010). Although there are shreds of evidence of the involvement of transcription factors in the trans-differentiation process of the MSCs into proliferative VSMCs (Hirschi and Majesky, 2004; Tamama et al., 2008). Besides, there is a lack of studies regarding the involvement of transcription factors during the transdifferentiation of HSCs into proliferative VSMCs. In this section, we have primarily focused on the hematopoietic origin of vascular and cardiac cells in response to different stimuli like ischemia, mechanical or inflammatory stress, and their contribution to CVDs.

### Bone Marrow-Derived Vascular Smooth Muscle Cells

Several studies have suggested a possibility of heterogeneous origins of the proliferative VSMCs in the atherosclerotic plaques. These heterogeneous origins range from bone marrow-derived HSCs to resident stem cell populations within the arterial wall (Bennett et al., 2016; Basatemur et al., 2019). However, studies involving atherosclerotic patients and atherosclerosis prone high-fat diet-fed ApoE^–/–^ and LDLR^–/–^ mice, have indicated the possibility of HSCs contributing to the plaque build-up and VSMC population. Evidence suggests that bone marrow-derived HSCs act as a source for most of the vascular cells in the neointima and media, and contribute to arterial remodeling in atherosclerotic patients and high-fat diet-fed ApoE^–/–^ mice (Sata et al., 2002). Studies have reported that ∼100 fold increase in the number of donor SMCs in the intima, media, and adventitial microvessels of atherosclerotic coronary arteries in recipients of a sex-mismatched bone marrow transplant (Caplice et al., 2003). A major population of the cells in the neointima of the coronary arteries and their smaller branches, collected post neointimal hyperplasia were found to be of recipient origin in mice which, underwent heterotopic cardiac transplantation from a donor mouse (Saiura et al., 2001). These results also indicated the existence of bone marrow-derived circulating SMC progenitor cells in the graft/damaged vessel intima which contributes to neointima formation. Contradictory reports regarding seldom contribution of HSCs to the vascular cells involved in vascular damage has also been published. In post-angioplasty restenosis, graft vasculopathy, and high-fat diet-induced atherosclerosis models, hematopoietic progenitor cells were shown to migrate to the injured vessel area and contribute to macrophage/inflammatory monocyte lineage (Iwata et al., 2010). The potential of different bone marrow fractions giving rise to the vascular cells which either repair or form the neointima in the arterial lesions has been delineated. The arterial lesions contained a significant number of vascular cells of bone marrow origin in mice which received total bone marrow fractions compared to those that received purified single-cell HSC population. This may suggest that *trans*-differentiation of HSC derived vascular cells may contribute less to the vascular cell in the neointima formation or its repair when compared to the whole bone marrow fraction having a varied population of cells (Sahara et al., 2005). Hence, the hematopoietic origin of vascular cells in vascular remodeling is still debated and requires more studies with rigorous lineage tracing methodologies.

### Circulating HSC Derived Endothelial Cells

There also have been similar reports of bone marrow HSC derived circulating EPC contributing to ECs to repair trauma-induced vascular intima damage in humans, mice, and rabbit models in the past (Bonnet, 2002). Patients with acute myocardial infarction have been shown to have a large number of circulating EPCs and CD34^+^ mononuclear cells post-infarction (Shintani et al., 2001). This may suggest that myocardial infarction potentially induces the bone marrow to mobilize EPCs and CD34^+^ mononuclear cells to repair the infarction induced vascular damage. The contribution of EPCs to neovascularization has also been shown well in the myocardial infarction rat model. Radiolabelled human EPCs transplanted into post-myocardial infarction in athymic nude rats were shown to be incorporated more in ischemic myocardium than non-ischemic myocardium (Aicher et al., 2003). Isolated human circulating CD34^+^Flk^+^ mononuclear cells have been shown to transform into EC-like phenotype *in vitro*. Additionally, CD34^+^Flk^+^ mononuclear cells isolated from heterologous (human), homologous (transgenic mice), and autologous sources were shown to contribute to the EC population involved in neoangiogenesis in mice and rabbit hind limb ischemia models (Asahara et al., 1997; Takahashi et al., 1999; Kalka et al., 2000; Bonnet, 2002). Similarly, in myocardial infarction rat models, injection of human CD34^+^ BM-derived EPCs followed by G-CSF administration has been shown to differentiate into ECs. These cells promote neoangiogenesis in the hypertrophied myocardium and thus improves the cardiac function (Kocher et al., 2001). In another study, BM-derived ECs were shown to repopulate the hepatic venous endothelium in the mouse bone marrow transplant model and patients receiving sex-mismatched liver transplant (Gao et al., 2001). Similarly, in patients receiving kidney transplants, bone marrow and the ECs of recipient origin have been found contributing to the repair damaged intima in the graft vessels but its association with graft rejection remains debatable (Sinclair, 1972; Andersen et al., 1991; Lagaaij et al., 2001; Poulsom et al., 2001; Bonnet, 2002). CD34^+^ EPCs isolated from human peripheral blood have also been shown to be of potential therapeutic use in myocardium infarction rat models by contributing to the post-infarction neovascularization and aiding to improve the associated symptoms (Shintani et al., 2006). On the other hand, the potential of BM-derived EPCs to contribute to the repair of dysfunctional/damaged endothelium in chronic vascular disease has been challenged by studies in different animal models. In one such study, EGFP^+^ eNOS^+^ BM cells were grafted into irradiated or busulfan injected eNOS^–/–^ mice. No EGFP^+^ eNOS^+^ ECs were detected in the intima of the coronary, hepatic, renal, and splenic arteries, as well as terminal arterioles in the heart, liver, spleen, and kidney of the recipient mice. This disputed the potential of BM-derived cells to contribute to the repair of dysfunctional endothelium in chronic vascular disease (Perry et al., 2009). Likewise, in another study, lethally irradiated mice that received BM transplants from CAG-EGFP transgenic mice were subjected to a dorsal excisional wound. However, no GFP^+^ cells were found in the EC population of the wound healing edge area suggesting that BM-derived cells do not contribute to the replacement of ECs in wound healing. This perivascular localization of BM-derived cells has also been supported by another study in mice that received VEGF inoculations or syngeneic B16 melanomas to induce angiogenesis. It was found that BM-derived cells do not contribute to the local EC population but to perivascular/periendothelial cell populations at angiogenic sites (Purhonen et al., 2008; Okuno et al., 2011). Similar results have also been reported in a study involving a transplant arteriosclerosis rat model where aortic allografting was performed on BM transplant recipient chimeric rats. Only 1–3% of the ECs in the graft neointima were found to be of bone marrow origin (Hillebrands et al., 2002). Several recent studies have also explored the role of endogenous vascular progenitor cells like tissue resident EPCs, c-kit^+^ stem/progenitor cells, endocardial cells and epicardial cells in contributing to the EC population during vasculogenesis as well as endothelial regeneration. Additionally, pre-existing ECs have also been shown to contribute to the neovascularization in coronary vessel post-injury (Kovacic et al., 2008; Xu, 2008; Zhang H. et al., 2016; He et al., 2017; Wörsdörfer et al., 2017; Chen et al., 2018; He and Zhou, 2018; Tang et al., 2018b; Zhang et al., 2018). Despite sharing a similar phenotype, these EPCs may have different biomarker profiles due to originating from different sources viz. bone marrow, MSCs myeloid cells, and other non-hematopoietic sources (Chopra et al., 2018). Hence, due to all these contradicting results and lack of widely adopted EPC specific markers, the contribution of BM HSC derived EPCs to the EC population involved in post-injury neovascularization is controversial (Patel and Donovan, 2012; Zhao et al., 2013; He et al., 2020a). Nevertheless, several studies have reported that BM-derived EPCs may play a potential role in designing therapeutic strategies to repair endothelial damage in vascular diseases, either via. their direct recruitment or EPC derived exosomes, microvesicles (Chen et al., 2011, 2012, 2013; Ranghino et al., 2012; Wang et al., 2013; Zhao et al., 2013). Currently, the potential role of the exosomes and/or microvesicles derived from HSCs, carrying transcription factors as cargo and modulating regenerative potential remains unexplored and may open new avenues of research.

### HSC Derived Cardiomyocytes

Initially, the process of cardiogenesis in adult hearts under normal- or pathophysiological conditions was attributed to the process of cardiac hypertrophy. Soon after that, evidence supporting the contribution of cardiac progenitor cells to the functional cardiomyocyte population during cardiac tissue regeneration was also reported. The origin of these cardiac progenitor cells and the degree of contribution, however, remains debatable. Several studies have reported HSCs along with MSCs, resident cardiac side-population (SP), and EPCs as potential sources of these cardiac progenitor cells (Jackson et al., 2001; Orlic et al., 2001a; Beltrami et al., 2003; Cornel et al., 2003; Oh et al., 2003; Planat-Bénard et al., 2004; Laugwitz et al., 2005; Otmar et al., 2005; Shiota et al., 2007; Reinecke et al., 2008; He et al., 2020b).

Continuous cardiomyocyte apoptosis and necrosis during hypoxia in acute myocardial infarction and during reperfusion ultimately leads to the fibrosis of the myocardium and decrease in cardiovascular function. CD34^+^ specific HSCs have been shown to transdifferentiate into all three major cell lineages of the cardiovascular system viz. cardiomyocytes, VSMCs, and ECs in coronary artery occlusion induced infarct model in SCID mice (Antti et al., 1997; Manzar et al., 1997; Anversa et al., 1998; Yeh et al., 2003). Upon transplantation of HSC enriched bone marrow subpopulation cells in mice that underwent coronary artery ligation, a small subpopulation of cardiomyocytes (∼0.02%) in the infarcted myocardium was found to be of donor origin. This indicates the ability of HSCs to transdifferentiate into cardiomyocyte like populations under pathophysiological conditions (Jackson et al., 2001). Another group investigated the contribution of bone marrow-derived HSCs to the myocardium. In wild-type mice, that underwent coronary artery ligation and subsequently received an injection of a Lin^–^ c-kit^+^ stem cell subpopulation from the bone marrow of a GFP expressing mice into the contracting wall bordering the infarct. A majority (∼68%) of the infarcted myocardium was found to be occupied by the proliferating cardiomyocytes of allograft origin (GFP expressing) along with newly formed vasculature. The same group later injected a combination of stem cell factor (SCF) and granulocyte-colony-stimulating factor (G-CSF) to mobilize injected GFP Lin^–^ c-kit^+^ BM stem cells in mice with ligated coronary artery and found similar results with improved regeneration and mortality (Orlic et al., 2001a,b). Other reports involving coronary artery ligation induced infarct model in SCID mice and NOD/SCID/IL2rγ^null^ mice have also shown the similar ability of CD34^+^ HSCs to contribute to host cardiomyocytes (Yeh et al., 2003; Fukata et al., 2013). On the other hand, studies with results contradicting the abovementioned conclusions have also been published. In coronary artery occlusion induced myocardial infarct mouse model and subsequent injection of mice with enriched HSCs, no donor origin cardiomyocytes in the infarct region of the myocardium were observed. This suggests the *trans*-differentiation of HSCs to cardiac lineage cells post-infarction and injection have not occurred in these mice (Murry et al., 2004). Thus, the ability of bone marrow-derived HSCs to contribute to cardiomyocyte population in pathophysiological conditions remains controversial. However, it should be noted that transdifferentiation of MSCs to cardiomyocytes *in vitro* via overexpression of GATA-4 has been reported. This suggests that GATA-4 and other transcription factors may play a similar role in the differentiation of BM-derived HSCs to cardiomyocytes (Li et al., 2011).

Additionally, the role of tissue-resident cardiac stem and progenitor cells in cardiac regeneration has also been a topic of debate over the years among researchers. C-kit^+^ stem cells and Sca^+^ resident progenitor cells in the adult heart have been shown to contribute to the SMC, cardiomyocyte, and EC populations to varying degrees. Some reports, on the other hand, have altogether refuted their contribution. However, it is beyond the scope of this review (Beltrami et al., 2003; Garry and Olson, 2006; Lyngbæk et al., 2007; Kehat and Molkentin, 2010; Ellison et al., 2013; Molkentin and Houser, 2013; Xin et al., 2013; Molkentin, 2014; Nadal-Ginard et al., 2014; Van Berlo et al., 2014; Van Berlo and Molkentin, 2014, 2016; Kanisicak et al., 2017; He et al., 2018; Maliken et al., 2018; Neidig et al., 2018; Tang et al., 2018a; Vagnozzi et al., 2018; Ni et al., 2019). It is a well-established fact that most of the progenitor cells require transcription factors for differentiation and proliferation. A recent study has reported that transcription factors like GATA family, MEF2C, NKX2.5, and TBX5, individually or in a combinatorial manner, induce the expression of cardiomyocyte-specific markers in the cardiac progenitor cells/resident cells (Al-Maqtari et al., 2017). Hence, there arises a need to further investigate the role of transcription factors in the differentiation of HSCs into non-blood lineage cells. There is an increase in the burden of evidence supporting the contribution of HSCs to cardiomyocyte, VSMC, and EC populations in CVDs and their origin (Figure 1). As the extent of their contribution to the cardiogenesis, neointima formation, and vessel wall repair remain debatable due to the lack of consensus among researchers. This has resulted in multiple reports with contradicting results due to a lack of lineage tracing studies with the challenges of using multiple lineage-specific markers extensively. Additionally, there is a need for studies focusing on the role of different transcription factors in the differentiation of HSCs into cardiac and vascular cells and thus, provides an opportunity for further research.

## Clinical Aspects

Annually, ∼17⋅8 million deaths (worldwide) were attributed to CVDs (Mensah and Mackay, 2009). One of the major factors that contribute to the high mortality rate is the cardiovascular tissue’s inability to regenerate the damaged cell population; post-injury in ischemic heart diseases that further exacerbates the condition and eventually leads to cardiac failure. Thus, stem cell transplantation in patients with myocardial infarction can be used as a therapeutic strategy to induce cardiac regeneration through the repopulation of damaged cardiac tissue (Wollert and Drexler, 2005). Stem cell-based therapy is well known and has been used with limited success over the last decade as a therapeutic strategy against ischemic heart disease. Many multipotent stem cells like EPCs, HSCs, cardiac stem cells, MSCs, and pluripotent stem cells such as embryonic stem cells and induced pluripotent stem cells (iPSCs) are known to contribute to the damaged myocardial cell population thus, regenerating the cardiac tissue (Mueller et al., 2018). Clinical studies have reported that bone marrow stem cell transplantation can efficiently improve cardiac functions in myocardial infarction (MI) patients (Vrtovec et al., 2013; Highlight, 2014). In EPCs based therapy, it has been reported that both the endogenous and injected EPCs inhibit the stress-induced oxidative burden on mature differentiated ECs. In-addition EPCs secrete growth factors like VEGF, stromal-derived factor-1 (SDF1) which efficiently protects against ischemia-induced damage by recruiting more EPCs and protecting the existing functional ECs (Yang et al., 2010). HSCs contribute to cardiac regeneration mainly by regulating angiogenesis and neovascularization by releasing paracrine factors. HSCs are known to release paracrine factors like VEGF, Ang1&2, PDGF-B, and insulin-like growth factor 1 (IGF1) leading to neovascularization at the injured sites (Lemcke et al., 2017). Studies have reported that inducing the expression of cardiac-specific transcription factors like GATA4, MEF2C, and NKX2.5 increases the potential of cardiogenic differentiation of MSCs (Burchfield and Dimmeler, 2008). The contribution of cardiac-transcription factors in the MSC differentiation to cardiomyocytes and ECs have been studied in detail by several groups (Singh et al., 2016; Lemcke et al., 2017; Heidel et al., 2020). However, the modulation of transcription factors in the conditional differentiation of HSCs is unexplored, and mapping these lineage-specific studies will provide profound insights into the differentiation of HSCs to cardiomyocytes. Upregulating the expression of GATA6 in undifferentiated mouse embryonic stem cells (mESCs) and pluripotent stem cells lead to efficient differentiation into cardiomyocytes (Yoon et al., 2018). Another recent study has revealed the ability of human pluripotent stem cells (hPSCs) (under controlled stimulation) to give rise to cardiac fibroblasts (CFs) in the presence of TGF-β and AngII (Zhang et al., 2019). These differentiated CFs can be efficiently used as a therapeutic cell source in the ischemia-induced injury of myocardial tissue to regenerate the damaged fibroblast. However, it should be noted that the use of stem cells in regenerating the damaged tissue can also be detrimental due to a potential risk of tumor formation and expression of malignancy-like markers in the patients, post-therapy (Joggerst and Hatzopoulos, 2009). Extracellular vesicle-based therapy has emerged in recent years as an alternate method with promising therapeutic potential. HSC derived exosomes are widely studied and carry mainly microRNAs which help in anti-apoptotic and pro-angiogenic responses, and cardiac regeneration (Radosinska and Bartekova, 2017). Mesenchymal stromal cell-derived exosomes have been reported to decrease oxidative stress and improve the survival of cardiomyocytes in the ischemia-reperfusion mouse model. Recent proteomic analyses have also revealed that exosomes carry numerous transcription factors that further regulate apoptosis, cellular proliferation, and survival in the recipient (Kore et al., 2019). In this review, we have described in detail, various HSC transcription factors, and their role in the repair of myocardial and vascular damage due to multiple cardiovascular complications. The EV-based therapy, although currently under scrutiny owing to its invasive nature, has shown therapeutic potential in animal models. Numerous approaches have been developed to overcome the associated limitations, and have been fairly successful. Further extensive research is required to explore the potential therapeutic role of exosomes as a cargo molecule for transporting transcription factors to a target site and the role of transcription factors carried by HSCs derived exosomes. This also presents researchers with an interesting opportunity to investigate the downstream signaling cascades in cardiomyocytes in which transcription factors from HSC derived exosomes have been delivered as well as the extent of the resultant cardio-protective effects.

## Concluding Remarks and Future Perspective

Organogenesis has been extensively investigated in both embryo and adults in animal models as well as humans. Over the years, novel factors and regulatory mechanisms involved in the process have been reported, and yet there remains a gap in our comprehensive understanding of the phenomenon. Due to the complex multi-factor regulatory nature of hematopoiesis and its systemic manifestation, we posit that it must be simultaneously considered in investigating any disease development, be it chronic acquired, hereditary, or inflammatory. Several transcription factors have been shown to interact amongst themselves and with other proteins such as epigenetic modifiers to create a dense spatiotemporal gene regulatory network. This presents researchers with an interesting opportunity to probe into novel interactions between epigenetic modifiers and hematopoietic transcription factors that regulate HSCs pool. Although the phenomenon of HSCs contributing to cell populations of cardiovascular lineage via. *trans*-differentiation has been observed in several studies involving animal models as well as in bone-marrow transplant recipients and cardiac allografts recipients, it remains a topic of non-consensus among researchers. The recent use of stem cell transplantation and EVs based therapies for cardiac tissue regeneration have also shown tremendous therapeutic potential as clinical treatment of ischemic heart disease. Even so, the therapeutic potential of exosomes carrying the HSC derived transcription factors remains mostly unexplored. Unraveling the interactions within the complex network of the aforementioned transcription factors with epigenetic modifiers and investigating their potential as therapeutic targets would pave the way for a “target-specific therapy” to treat ischemic heart disease.

## Author Contributions

SD, RC, and AG collected the articles and analyzed and wrote the manuscript. PS supervised the project. All the authors contributed to the article and approved the submitted version.

## Conflict of Interest

The authors declare that the research was conducted in the absence of any commercial or financial relationships that could be construed as a potential conflict of interest.

## Abbreviations

CVDs: cardiovascular diseases
ECs: endothelial cells
EPCs: endothelial progenitor cells
HSCs: hematopoietic stem cells
HUVECs: human umbilical vein endothelial cell
SMCs: smooth muscle cells
VSMCs: vascular smooth muscle cells.
